# Efficacy of a hybrid assistive limb in post-stroke hemiplegic patients: a preliminary report

**DOI:** 10.1186/1471-2377-11-116

**Published:** 2011-09-27

**Authors:** Shinichiro Maeshima, Aiko Osawa, Daisuke Nishio, Yoshitake Hirano, Koji Takeda, Hiroshi Kigawa, Yoshiyuki Sankai

**Affiliations:** 1 Rehabilitation Medicine, Saitama Medical University International Medical Center, Hidaka, Japan; 2 Rehabilitation Center, Hanno Seiwa Hospital, Hanno, Japan; 3 Graduate School of Systems and Information Engineering, University of Tsukuba, Tsukuba, Japan

## Abstract

**Background:**

Robotic devices are expected to be widely used in various applications including support for the independent mobility of the elderly with muscle weakness and people with impaired motor function as well as support for nursing care that involves heavy laborious work. We evaluated the effects of a hybrid assistive limb robot suit on the gait of stroke patients undergoing rehabilitation.

**Methods:**

The study group comprised 16 stroke patients with severe hemiplegia. All patients underwent gait training. Four patients required assistance, and 12 needed supervision while walking. The stride length, walking speed and physiological cost index on wearing the hybrid assistive limb suit and a knee-ankle-foot orthosis were compared.

**Results:**

The hybrid assistive limb suit increased the stride length and walking speed in 4 of 16 patients. The patients whose walking speed decreased on wearing the hybrid assistive limb suit either had not received sufficient gait training or had an established gait pattern with a knee-ankle-foot orthosis using a quad cane. The physiological cost index increased after wearing the hybrid assistive limb suit in 12 patients, but removal of the suit led to a decrease in the physiological cost index values to equivalent levels prior to the use of the suit.

**Conclusions:**

Although the hybrid assistive limb suit is not useful for all hemiplegic patients, it may increase the walking speed and affect the walking ability. Further investigation would clarify its indication for the possibility of gait training.

## Background

**Figure 1:**
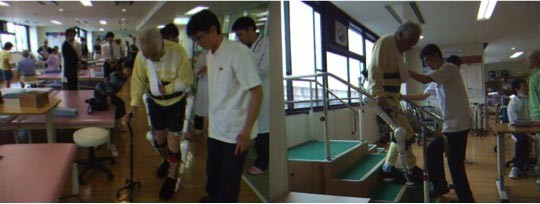
**A stroke patient using the hybrid assistive limb (HAL) suit**. The left image shows a patient using the HAL suit and walking with a cane, and a physiotherapist pays attention to the patient while noting his fall. In the right image, the patient is being trained to climb down stairs by a physiotherapist.

The issue of nursing care in an ageing society is a major social concern and will continue to be so in future. Therefore, robotic devices are expected to be widely used in various applications including support for the independent mobility of the elderly with muscle weakness and people with impaired motor function as well as support for nursing care that involves heavy laborious work [1-3]. The hybrid assistive limb (HAL) suit does not merely use the concept of power assist; it is a hybrid system composed of a 'cybernics voluntary control system' that provides complete control of the HAL suit using bioelectric signals and a 'cybernics robotic autonomous control system' that autonomously generates motor patterns reflecting characteristics of human motion (Figure 1). It is the world's first cyborg-type robot that takes advantage of cybernics that integrate human, mechanical and information technologies [4]. The leg structure of the HAL exoskeleton powers the flexion/extension joints at the hip and knee through a DC motor with a harmonic drive placed directly on the joints. The ankle flexion/extension degree of freedom is passive. Lower limb components interface with the wearer through a number of connections that include a special shoe with ground reaction force sensors, harnesses on the calf and thigh and a wide waist belt. The HAL system utilises several sensing modalities for control: skin-surface electromyographic (EMG) electrodes placed below the hip and above the knee on the anterior (front) and posterior (back) sides of the wearer's body, potentiometers and a gyroscope and accelerometer mounted on the backpack for torso posture estimation. These sensing modalities are in-built and operate the suit, which consists of EMG-based and walking pattern-based systems. The HAL suit was designed to increase and assist the voluntary motor fuction of the body and is used to provide walking support for people who require nursing care, including the elderly with muscle weakness and people with impaired motor function [4,5].

In Japan, stroke patients often require nursing care for the resulting hemiplegia and ataxia, which make walking difficult [6,7]. While attempts have been made to recover walking ability, there are few studies on the rehabilitation of hemiplegic patients [8]. In this study, we evaluated the effects of the HAL suit on the gait of stroke patients undergoing rehabilitation.

## Methods

The study group comprised 16 post-stroke hemiplegic patients (9 men and 7 women) who were admitted for rehabilitation with the goal of allowing them to return home (Table 1). The patients were aged between 53-78 years. Stroke was caused by cerebral infarcts in 7 patients, cerebral bleeding in 7 patients and subarachnoid haemorrhage in 2 patients. All patients had severe hemiplegia and were undergoing gait training with a long knee-ankle-foot orthosis (KAFO) [9,10]. Four patients with muscle weakness in the trunk and extremities required assistance to walk. The remaining 12 patients needed supervision during walking. Of these, 3 patients were able to walk while grasping the handrail, and 9 could walk using a quad cane. We then divided the patients into 3 groups based on the ability of ambulation: an assisted group, in which 4 patients with muscle weakness in the trunk and extremities required assistance to walk; a handrail group, in which 3 patients were able to walk using the handrail and a quad cane group, in which 9 patients were able to walk using a quad cane. The stride lengths, walking speeds and physiological costs were compared between the 3 groups.

**Table 1:** Clinical data of the subjects

Case	Age Gender	Etiology	Duration since stroke onset (days)	Duration since admission (days)	Duration since ambulation ex.(days)	Neurological deficits (BRS)	mAS	RMI	FAC	Cane	Group
1	75 M	CI	44	5	1	Lt-hemiplegia (3)	1	5	1	handrail	A
						neglect					
2	78 M	ICH	33	7	1	Rt-hemiplegia (3)	1+	5	1	handrail	A
						aphasia					
3	68 F	CI	44	9	1	Rt-hemiplegia (2)	2	4	1	handrail	A
						aphasia					
4	62 M	ICH	87	44	1	Rt-hemiplegia (2)	2	4	1	handrail	A
						aphasia					
5	70 M	CI	116	56	31	Lt-hemiplegia (2)	2	6	2	handrail	B
						neglect					
6	65 M	ICH	38	7	10	Lt-hemiplegia (3)	1	6	2	handrail	B
						neglect					
7	66 M	ICH	27	11	7	Lt-hemiplegia (3)	1	6	2	handrail	B
8	64 M	CI	48	19	17	Rt-hemiplegia (2)	2	6	2	Quad cane	C
						aphasia					
9	50 F	CI	38	9	6	Lt-hemiplegia (3)	1	6	2	Quad cane	C
10	63 F	SAH	47	9	5	Lt-hemiplegia (3)	1	6	2	Quad cane	C
11	55 F	SAH	53	13	9	Rt-hemiplegia (3)	1+	6	2	Quad cane	C
12	77 F	ICH	29	16	46	Lt-hemiplegia (3)	1	6	2	Quad cane	C
13	53 M	CI	42	24	20	Rt-hemiplegia (2)	1	5	2	Quad cane	C
14	53 M	CI	109	53	35	Lt-hemiplegia (3)	2	6	2	Quad cane	C
						neglect					
15	48 F	ICH	38	15	14	Lt-hemiplegia (3)	1	6	2	Quad cane	C
16	68 F	ICH	37	15	14	Rt-hemiplegia (3)	1	6	2	Quad cane	C
						aphasia					

BRS, Brunnstrom recovery stage; mAS, modified Asthworth scale; RMI, Rivermead mobility index; FAC, functional ambulation categories; M, male; F, female; CI, cerebral infarction; ICH, intracerebral hemorrhage; SAH, subarachnoid hemorrhage

Groups A, Required assistance during walking with handrail; Group B: Supervision during walking with handrail; Group C: Supervision during walking with quad cane

### Procedure

HAL is an exoskeleton robot that enhances and strengthens the limb motion of the human body by detecting the weak bioelectrical nerve signals sent by the brain to control the musculoskeletal system. It can be controlled for the degree of support for hip and knee flexion and extension. Physiotherapists place the electrodes on the patients' lumbar and lower limbs and confirm suit operation controlled with a computer. The settings were determined according to the severity of muscle contraction for each patient so that the patients could easily raise their lower extremities [11]. After standing up motions were repeated while considering patient safety, walking training was begun. In Figure 1, the left image shows a patient using the HAL suit and walking with a cane, and a physiotherapist pays attention to the patient while noting his fall. In the right image, the patient is being trained to climb down stairs by a physiotherapist.

### Evaluation

The patients wore the bilateral HAL suit and KAFO; their stride lengths and walking speeds using each support device were measured while they walked 10 m grasping a handrail. Moreover, the physiological cost index (PCI) was calculated to determine the gait efficiency [12]. The walking assessment was conducted at 4 time points: walking with the KAFO before wearing the HAL suit, walking with the HAL suit, walking with the KAFO after removing the HAL suit and walking with the KAFO the next day. Compared to walking with the KAFO before wearing the HAL suit, the stride length, walking speed and PCI were considered to be affected if increases or decreases of greater than 10% were observed. Data were statistically analysed using the two-way repeated measures analysis of variance (ANOVA).

The process of consent was in accordance with the Declaration of Helsinki and was approved by the institutional ethics committee (Hanno Seiwa Hospital, IRB No. 100106). All patients gave their written informed consent for study participation. Patients were made aware of their right to withdraw from the study at any time without adverse effects on their clinical care.

## Results

**Figure 2:**
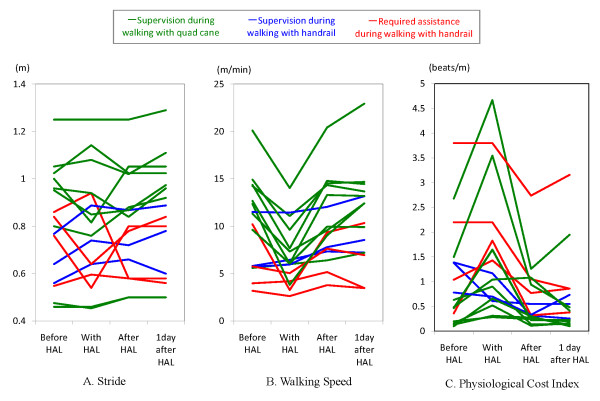
**Changes in the stride, walking speed and physiological cost index (PCI) before and after HAL use**. The HAL suit increased the stride length in 4 of 16 patients (A). The walking speed increased in 4 of 16 patients while walking with the HAL suit, and this change persisted even while walking with the KAFO after removing the HAL suit on the following day. The walking speed markedly decreased in 12 patients while wearing the HAL suit (B). The PCI values clearly decreased after wearing the HAL suit in only 2 patients. In 11 patients, the PCI values apparently increased after wearing the HAL suit, but removal of the HAL suit led to a decrease in PCI values in 8 of 12 patients to levels equivalent to or below those observed before wearing the HAL suit (C).

Figure 2 shows the changes in the stride length, walking speed and PCI calculated from the 10-m walks. The HAL suit increased the stride length in 4 of 16 patients. All three patients who required supervision when walking with a handrail showed increases in the stride length and gait velocity. One patient in a quad cane group showed an increase in the stride length, and 1 patient in the assisted group showed increased gait velocity.

Of the patients who showed increases in stride length, 2 showed a decrease in stride length after removing the HAL suit, and the other 2 patients showed a decrease in stride length on the day after wearing the HAL suit. Two-way repeated measures ANOVA was performed to investigate the relationship between improvement of the stride length and the ability of ambulation. The main effects of stride length administration (F = 0.98, df = 3) and the ability of ambulation (F = 1.33, df = 2) were not significant.

The walking speed increased in 4 of 16 patients while walking with the HAL suit, and this change persisted even while walking with the KAFO after removing the HAL suit on the following day. The walking speed markedly decreased in 12 patients while wearing the HAL suit. Four of these patients had not received sufficient gait training and required assistance in walking. The 8 other patients needed supervision and had to walk with a quad cane. Immediate improvement in walking was observed for 11 of 12 patients after removing the HAL suit, and, except for one patient, their walking speed improved with the use of the KAFO on the day after the removal of the HAL suit compared to that with the use of the KAFO before wearing the HAL suit. Two-way repeated measures ANOVA was performed to investigate the relationship between improvement of the walking speed and the ability of ambulation. The main effects of walking speed administration (F = 10.35, df = 3, *P *< .005) and the ability of ambulation (F = 4.88, df = 2, *P *< .05) were significant, as was the interaction between the walking speed and the ability of ambulation (F = 3.65, df = 3, *P *< .05).

The PCI values clearly decreased in only 2 patients after wearing the HAL suit. In 11 patients, the PCI values apparently increased after wearing the HAL suit, but removal of the HAL suit led to a decrease in PCI values in 8 of 12 patients to levels equivalent to or below those observed before wearing the HAL suit. Finally, PCI values recovered while walking with the KAFO on the day after wearing the HAL suit in other patients compared to those while walking with the KAFO before wearing the HAL suit. Two-way repeated measures ANOVA was performed to investigate the relationship between improvement of the PCI values and the ability of ambulation. The main effects of PCI administration (F = 3.24, df = 3) and the ability of ambulation (F = 1.63, df = 2) were not significant.

## Discussion

Recent evidence indicates the advantages of combining robotics with existing training programs to enhance the functional improvement of movement tasks for people with neurological disorders [1-3,13-15]. Most robotic technology applied to rehabilitation allows passive gait exercise on a treadmill [2,15,16]. Conversely, the HAL suit is an exoskeleton robot that provides the possibility of assistive continuous movement within the electric reception area of a personal computer [4,8]. The HAL suit uses bioelectric signals to provide power assistance, which may make it difficult for severely hemiplegic patients to perform activities using their muscles, as observed in our study. This could lead to instability, resulting in decreases in stride length and walking speed. Walking while wearing the HAL suit requires adaptation to a new gait pattern and coordination of movements. In contrast to healthy people, severely hemiplegic patients may experience difficulty in rapidly adapting to a new gait pattern. Particularly, the walking speed of all 4 patients requiring assistance in our study markedly decreased while wearing the HAL suit because of muscle weakness of the trunk and extremities. These results suggest that the HAL suit may not be suitable for patients without sufficient gait training because it may increase the difficulty of walking in such patients. Furthermore, the patients who needed supervision during walking and use of a quad cane and those who had an established gait pattern to a certain degree demonstrated a significantly disturbed gait pattern after wearing the HAL suit.

On the other hand, all 3 patients who needed supervision while walking and had to grasp the handrail before wearing the HAL suit mastered an efficient gait pattern, as evidenced by increases in their stride length and walking speed. In addition, decreases in their PCI values after wearing the HAL suit were observed. We therefore conclude that the HAL suit may increase the walking speed and stride length and could be used for gait training; however, further investigation is necessary to determine the patients most likely to benefit from use of the HAL suit. For proper use of the suit, it is desirable for severely hemiplegic patients to have muscular strength in healthy lower extremities and trunk to allow walking. Furthermore, it is advisable that patients have already received gait training with a long KAFO before wearing the HAL suit. If a patient's paralysis is so severe that it causes muscle contraction or his/her bioelectric signals cannot be sensed, the HAL suit should not be used. In addition, our results suggest that even patients who have undergone sufficient gait training and have established their own new gait pattern may not benefit from the use of a HAL suit. Nevertheless, it is noteworthy that, although the stride length and walking speed results varied among patients, none showed an increase in PCI values, and 6 patients could walk more efficiently with a KAFO on the day after wearing the HAL suit compared with walking with a KAFO before wearing the HAL suit. The use of the HAL suit forced the patients and their therapists to pay more attention to several factors involved in walking such as raising the lower extremities, stride length and walking speed. On the other hand, the use of the HAL suit in patients with severe hemiplegia may contribute not only to improved walking while wearing the HAL suit but also to the learning involved in the adoption of a new gait pattern while walking after removal of the HAL suit.

## Conclusion

The HAL suit is expected to be widely used in gait training. Further study is warranted to establish the efficacy of its continuous use and determine the indications for using the HAL suit.

## Competing interests

The authors declare that they have no competing interests.

## Authors' contributions

SM drafted the manuscript. AO participated in the study design and helped to draft the manuscript. DN participated in the study coordination and conducted physical therapy programs. YH and KT conducted physical therapy programs. HK participated in the medical examination. YS participated as a developer of robotics. All authors read and approved the final manuscript.

## Pre-publication history

The pre-publication history for this paper can be accessed here:

http://www.biomedcentral.com/1471-2377/11/116/prepub

## References

[B1] Volpe BT Krebs HI Hogan N Edelstein L Diels C Aisen M A novel approach to stroke rehabilitation. Robot-aided sensorimotor stimulation Neurology 2000 54 1938 1944 10822433 10.1212/wnl.54.10.1938

[B2] Hidler J Nichols D Pelliccio M Brady K Advances in the understanding and treatment of stroke impairment using robotic devices Top Stroke Rehabil 2005 12 22 35 10.1310/RYT5-62N4-CTVX-8JTE 15940582

[B3] Banala SK Kim SH Agrawal SK Scholz JP Robot assisted gait training with active leg exosketon (ALEX) IEEE Trans Neural Syst Rehabil Eng 2009 17 2 8 19211317 10.1109/TNSRE.2008.2008280

[B4] Kawamoto H Sankai Y Power assist method based on phase sequence and muscle force condition for HAL Adv Robot 2005 19 717 734 10.1163/1568553054455103

[B5] Lee S Sankai Y Virtual impedance adjustment in unconstrained motion for an exoskeletal robot assisting the lower limb Adv Robot 2005 19 773 795 10.1163/1568553054455095

[B6] Shimizu H Kawarai T Saji N Tadano M Kita Y Tabuchi M Re-evaluation of clinical features and risk factors of acute ischemic stroke in Japanese longevity society Kobe J Med Sci 2009 55 E132 E139 20847601

[B7] Muraki I Yamagishi K Ito Y Fujieda T Ishikawa Y Miyagawa Y Caregiver burden for impaired elderly japanese with prevalent stroke and dementia under long-term care insurance system Cerebrovasc Dis 2008 25 234 240 10.1159/000113861 18216465

[B8] Kawamoto H Hayashi T Sakurai T Eguchi K Sankai Y Development of single leg version of HAL for hemiplegia Conf Proc IEEE Eng Med Biol Soc 2009 2009 5038 5043 19964376 10.1109/IEMBS.2009.5333698

[B9] Maeshima S Ueyoshi A Osawa A Ishida K Kunimoto K Shimamoto Y Matsumoto T Mobility and muscle strength contralateral to hemiplegia from stroke: Benefit of self-training with family support Am J Phys Med Rehabil 2003 82 456 462 12820789

[B10] Yamanaka T Akashi K Ishii M Stroke rehabilitation and long leg brace Top Stroke Rehabil 2004 11 6 8 10.1310/G8RF-312L-G6FW-A8JW 15480948

[B11] Finley FR Cody KA Locomotive characteristics of urban pedestrians Arch Phys Med Rehabil 1970 51 423 426 5433607

[B12] MacGregor J Stoot FD, Raftery EB, Goulding L The objective measurement of physical performance with long term ambulatory physiological surveillance equipment (LAPSE) Proceedings of 3rd international symposium on ambulatory monitoring 1979 London: Academic Press 29 39

[B13] Krebs HI Hogan N Aisen ML Volpe BT Robot-aided neurorehabilitation IEEE Trans Rehabil Eng 1998 6 75 87 10.1109/86.662623 9535526 PMC2692541

[B14] Daly JJ Hogan N Perepezko EM Krebs HI Rogers JM Goyal KS Response to upper-limb robotics and functional neuromuscular stimulation following stroke J Rehabil Res Dev 2005 42 723 736 10.1682/JRRD.2005.02.0048 16680610

[B15] Hachisuka K Robot-aided training in rehabilitation Brain Nerve 2010 62 133 140 20192033

[B16] Hornby TG Campbell DD Kahn JH Demott T Moore JL Roth HR Enhanced gait-related improvements after therapist-versus robotic-assisted locomotor training in subjects with chronic stroke. A randomized controlled study Stroke 2008 39 1786 1792 10.1161/STROKEAHA.107.504779 18467648

